# Teachers’ emotional support and college students’ adaptation: the mediating roles of intrinsic goals and psychological need satisfaction

**DOI:** 10.3389/fpsyg.2026.1792547

**Published:** 2026-04-10

**Authors:** Ding Bing

**Affiliations:** 1 Shaanxi Institute of Teacher Development, Shaanxi Normal University, Xi’an, China; 2 Ankang University, Ankang, China

**Keywords:** chain mediation, college students’ adaptation, intrinsic goals, psychological need satisfaction, Self-Determination Theory, teachers’ emotional support

## Abstract

**Introduction:**

This study investigates the mechanisms through which teachers’ emotional support influences college students’ adaptation, focusing on the chain mediating roles of intrinsic goals and psychological need satisfaction, grounded in Self-Determination Theory (SDT).

**Methods:**

A stratified cluster sampling approach was used to collect data from 632 undergraduate students across China. Confirmatory Factor Analysis (CFA) was conducted for construct validity, and mediation analyses were performed using Hayes’ PROCESS macro (Model 6).

**Results:**

(1) Teachers’ emotional support significantly and positively predicts college students’ adaptation (*β* = 0.48, *p* < 0.001); (2) the direct effect of intrinsic goals on adaptation is non-significant (p=0.086), indicating intrinsic goals influence adaptation indirectly via psychological need satisfaction; (3) the chain mediation pathway is statistically significant (95% CI [0.034, 0.074]), with psychological need satisfaction accounting for 78.9% of the total indirect effect.

**Discussion:**

These findings provide empirical evidence for the internalization process of external support, suggesting teachers’ emotional scaffolding primarily aids adaptation by fostering intrinsic aspirations that subsequently fulfill students’ basic psychological needs.

## 1 Introduction

### 1.1 Research background

The transition to college represents a critical developmental period marked by significant academic, social, and psychological challenges. College students must navigate new learning environments, establish peer relationships, develop autonomy, and manage increased responsibilities. Successful adaptation to these challenges is crucial for students’ academic achievement, psychological well-being, and future career development.

Recent research indicates that college students’ adaptation difficulties have become increasingly prevalent, with studies documenting rising rates of psychological distress, academic struggles, and social isolation among undergraduate populations (Alvarez, 2023). The “sorrow of transition” phenomenon highlights the vulnerability of college students during this developmental phase, underscoring the need for understanding factors that promote successful adaptation.

Among the various factors influencing college students’ adaptation, social support from significant others plays a fundamental role (Cohen et al., 1985). While peer support and family support have received considerable attention, the role of teachers in supporting students’ adaptation deserves further investigation. In the higher education context, teachers are not merely knowledge transmitters but also serve as mentors, role models, and sources of emotional support.

However, several research gaps remain. First, while general social support has been studied, the unique influence of teachers’ emotional support in the higher education context—where students transition toward professional independence—is often overlooked. Second, existing studies frequently focus on direct associations, such as the study on the impact of social support on college students’ goal pursuit (Chen et al., 2022) and the research on the positive correlation between social support and a sense of life meaning (Jingxue Cai, 2022), leaving the internal psychological mechanisms (i.e., how external support translates into internal goal orientation and subsequent need satisfaction) insufficiently explored. This study addresses these gaps by integrating Goal Content Theory and Self-Determination Theory into a chain mediation model.

Teachers’ emotional support—characterized by warmth, caring, empathy, and responsiveness to students’ emotional needs—has been identified as a crucial factor in students’ academic engagement, motivation, and psychological well-being (Jia, 2012). However, the mechanisms through which teachers’ emotional support influences college students’ adaptation remain insufficiently understood. Clarifying these mechanisms is essential for developing effective educational interventions and informing teacher training programs.

### 1.2 Theoretical framework

This study draws on Self-Determination Theory (SDT) to explain the mechanism linking teachers’ emotional support to college students’ adaptation (Deci and Ryan, 1985, 2000). SDT posits that human beings have three basic psychological needs: autonomy (the need to feel volitional and self-directed), competence (the need to feel effective in one’s interactions with the environment), and relatedness (the need to feel connected to others). The satisfaction of these needs is essential for optimal functioning, well-being, and adaptive development (Vansteenkiste et al., 2020).

According to SDT, the social environment plays a critical role in either supporting or thwarting psychological need satisfaction. Supportive social contexts that provide autonomy support, structure, and involvement facilitate need satisfaction and promote autonomous motivation, well-being, and adaptive outcomes. Conversely, controlling or neglectful environments undermine need satisfaction and lead to maladaptive outcomes (Deci et al., 2008).

Teachers’ emotional support represents a key aspect of the supportive social environment in educational settings. When teachers demonstrate genuine care, empathy, and responsiveness to students’ emotional needs, they create a sense of safety and belonging that facilitates students’ exploration, learning, and growth. This supportive context is likely to promote psychological need satisfaction by enhancing students’ sense of relatedness, supporting their autonomy, and fostering their competence development.

Goal Content Theory (GCT), a mini-theory within SDT, further elaborates the distinction between intrinsic and extrinsic goals (Deci and Ryan, 2000; Vansteenkiste et al., 2006). Intrinsic goals—such as personal growth, meaningful relationships, and community contribution—are inherently satisfying and align with psychological need satisfaction. Extrinsic goals—such as wealth, fame, and image—are focused on external rewards and social approval and are less conducive to need satisfaction and well-being. Intrinsic goals (personal growth, affiliation) provide direct satisfaction of basic needs, whereas extrinsic goals (wealth, fame) may distract from or even thwart these needs.

Research has demonstrated that the pursuit of intrinsic goals is associated with greater psychological need satisfaction, well-being, and adaptive functioning, whereas the pursuit of extrinsic goals is associated with poorer outcomes (Kasser and Ryan, 1996; Vansteenkiste et al., 2006). In the educational context, students who pursue intrinsic academic goals (e.g., mastering knowledge, developing skills) tend to experience greater engagement, persistence, and academic success compared to those who pursue extrinsic goals (e.g., grades, recognition) (Li and Feng, 2018; Ye, 2017).

### 1.3 Literature review and hypothesis development

#### 1.3.1 Teachers’ emotional support and college students’ adaptation

Social support theory posits that supportive relationships buffer the negative effects of stress and promote adaptive coping (Cohen et al., 1985). In the college context, teachers represent significant social support providers who can help students navigate academic challenges, develop coping strategies, and maintain psychological well-being.

Empirical studies have consistently demonstrated positive associations between teacher support and student adaptation outcomes. Ying et al. (2017) found that undergraduate supervisor guidance positively predicted freshmen’s college adaptation, with self-efficacy serving as a mediating mechanism. Similarly, Yu et al. (2024) reported that social support was significantly associated with psychological well-being among UK university students.

Specifically, teachers’ emotional support—encompassing warmth, empathy, and responsiveness—creates a secure base from which students can explore new environments, take academic risks, and develop resilience in the face of challenges. The emotional dimension of teacher support may be particularly important during the transition to college, when students are separated from familiar support networks and must establish new relationships and identities.

Based on the theoretical framework and empirical evidence, we propose:

*Hypothesis* 1: Teachers' emotional support positively predicts college students’ adaptation.

#### 1.3.2 The mediating role of intrinsic goals

According to Goal Content Theory, the goals that individuals pursue have significant implications for their motivation, well-being, and adaptation (Deci and Ryan, 2000). Intrinsic goals—oriented toward personal growth, relationships, and community—are inherently satisfying and promote autonomous motivation. Extrinsic goals—oriented toward wealth, fame, and image—are contingent on external validation and are associated with controlled motivation.

According to the “organismic integration” process within SDT, the internalization of values largely depends on the quality of the interpersonal environment. When teachers provide high levels of emotional support—characterized by empathy, non-judgmental listening, and unconditional positive regard—they reduce students’ defensive reliance on external validation (such as GPA rankings or peer approval). Instead of feeling pressured to pursue extrinsic goals to prove their worth, emotionally supported students feel safe enough to explore their genuine interests. This secure emotional base catalyzes a shift in motivation, allowing students to internalize the teacher’s supportive values and adopt intrinsic goals centered on self-improvement, deep learning, and meaningful social contributions. Thus, teachers’ emotional support acts as an interpersonal catalyst that shifts students’ motivational orientation from external contingencies to intrinsic aspirations.

Research supports the link between supportive social environments and intrinsic goal pursuit. Lekes et al. (2010) found that parental autonomy support was associated with intrinsic life goals among adolescents in China and North America. Similarly, studies have shown that autonomy-supportive teaching practices promote students’ intrinsic motivation and mastery goal orientations.

Furthermore, intrinsic goal pursuit has been linked to adaptive outcomes. Students who pursue intrinsic goals tend to experience greater academic engagement, persistence, and achievement (Vansteenkiste et al., 2006). Ye, (2017) found that intrinsic goal content was associated with greater subjective well-being among Chinese college students, with basic psychological need satisfaction mediating this relationship.

Therefore, we propose:

*Hypothesis* 2: Intrinsic goals mediate the relationship between teachers' emotional support and college students’ adaptation.

#### 1.3.3 The mediating role of psychological need satisfaction

Self-Determination Theory posits that psychological need satisfaction is a fundamental mechanism through which social environments influence well-being and adaptive functioning (Vansteenkiste et al., 2020). When individuals experience satisfaction of their needs for autonomy, competence, and relatedness, they are more likely to function effectively, experience positive emotions, and adapt successfully to environmental demands.

Teachers’ emotional support is theoretically linked to psychological need satisfaction through multiple pathways. First, emotional support directly addresses students’ need for relatedness by providing a sense of connection, belonging, and being cared for. Second, supportive teachers who demonstrate respect for students’ perspectives and choices promote autonomy satisfaction. Third, teachers who provide encouragement, constructive feedback, and scaffolding support students’ competence development (Garn et al., 2018).

Empirical research supports the association between teacher support and psychological need satisfaction. Studies have shown that autonomy-supportive teaching practices predict greater need satisfaction among students (Gunnell et al., 2014). Furthermore, need satisfaction has been consistently linked to positive academic and psychological outcomes, including academic achievement, well-being, and adaptive coping.

Therefore, we propose:

*Hypothesis* 3: Psychological need satisfaction mediates the relationship between teachers’ emotional support and college students' adaptation.

#### 1.3.4 The chain mediation model

Integrating the preceding hypotheses, we propose a chain mediation model in which teachers’ emotional support influences college students’ adaptation through the sequential mediation of intrinsic goals and psychological need satisfaction. According to this model, teachers’ emotional support first promotes students’ intrinsic goal orientation, which in turn facilitates psychological need satisfaction, ultimately leading to better adaptation outcomes.

**Figure 1:**
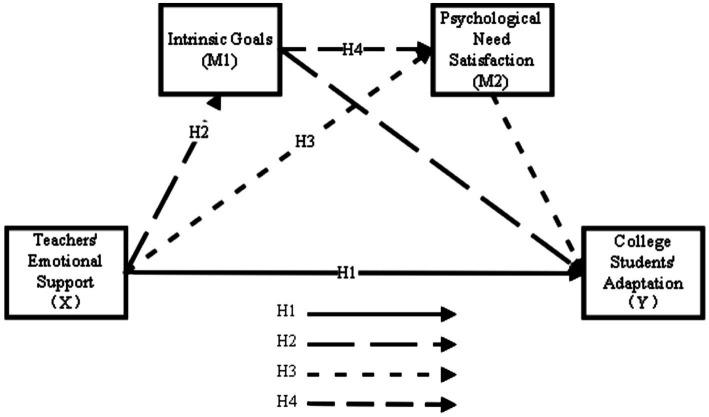
Hypothesized model.

This sequential process is consistent with SDT’s emphasis on the interplay between goal content and need satisfaction. Research has demonstrated that intrinsic goal pursuit is associated with greater psychological need satisfaction, which in turn predicts well-being and adaptive outcomes (Gunnell et al., 2014). The chain mediation model captures this theoretical logic by specifying the temporal and causal sequence from environmental support to goals to need satisfaction to adaptation (see Figure 1).

Therefore, we propose:

*Hypothesis* 4: Intrinsic goals and psychological need satisfaction sequentially mediate the relationship between teachers' emotional support and college students' adaptation (chain mediation).

### 1.4 Research contributions

This study makes several contributions to the literature. First, it extends Self-Determination Theory to the higher education context by examining how teachers’ emotional support influences college students’ adaptation through intrinsic goals and psychological need satisfaction. While SDT has been extensively applied in educational research, relatively few studies have examined these processes specifically among college students in China.

Second, this study provides a comprehensive examination of the mechanisms linking teacher support to student adaptation. By testing a chain mediation model, we clarify not only whether but also how teachers’ emotional support influences adaptation outcomes. This mechanistic understanding is crucial for developing targeted interventions.

Third, this study contributes to the growing literature on college student adaptation in China. As Chinese higher education continues to expand and diversify, understanding factors that promote successful adaptation is increasingly important for educational policy and practice.

## 2 Method

### 2.1 Participants and procedure

This study employed a cross-sectional survey design. To ensure sample representativeness, data were collected using a stratified cluster random sampling approach rather than simple random sampling. The stratification was primarily based on university tiers (elite “Double First-Class” universities, ordinary public universities, and private institutions) and geographic regions (Eastern, Central, and Western China). Within each selected institution, classes were randomly selected across different academic years (freshmen to seniors), and the online survey link was distributed to these specific clusters by institutional counselors. A total of 700 questionnaires were initially collected. After screening for completion time (excluding responses under 120 s) and careless response patterns (e.g., straight-lining), 632 valid responses were retained, yielding an effective response rate of 90.3%.

The sample composition was characterized by diverse institutional representation and balanced demographics. Regarding institution type, 416 students (65.82%) were from Double First-Class universities, 144 (22.78%) from ordinary public undergraduate institutions, and 72 (11.39%) from private undergraduate institutions. In terms of academic distribution, the sample included 219 freshmen (34.65%), 189 sophomores (29.91%), 127 juniors (20.09%), and 97 seniors (15.35%). Gender distribution was relatively equal, with 325 males (51.42%) and 307 females (48.58%). Additionally, the residential backgrounds of participants were almost evenly split between rural (*n* = 310, 49.05%) and urban (*n* = 322, 50.95%) areas.

Participation was voluntary, and informed consent was obtained from all participants. The study received ethical approval from the institutional review board. Participants were assured of confidentiality and could withdraw at any time without penalty.

### 2.2 Measures

#### 2.2.1 Teachers’ emotional support

Teachers’ emotional support was measured using the Teachers’ Emotional Support Scale developed by Jia (2012). This scale consists of 23 items assessing students’ perceptions of their teachers’ warmth, caring, empathy, and responsiveness to their emotional needs. Sample items include “My teachers show genuine concern for my well-being” and “My teachers are understanding when I experience difficulties.” Items were rated on a 5-point Likert scale ranging from 1 (strongly disagree) to 5 (strongly agree). The scale demonstrated good internal consistency in the present study (Cronbach’s *α* = 0.87).

#### 2.2.2 Intrinsic goals

Intrinsic goals were measured using the Goal Content Scale, a Chinese adaptation of the Aspiration Index validated by Tang et al. (2008). This scale assesses the importance individuals place on various life goals, distinguishing between intrinsic goals (personal growth, meaningful relationships, community contribution) and extrinsic goals (wealth, fame, image). For the present study, we focused on the intrinsic goal subscale, which comprises 20 items. Sample items include “It is important to me to grow and learn new things” and “It is important to me to have deep, meaningful relationships.” Items were rated on a 7-point Likert scale ranging from 1 (not at all important) to 7 (very important). The intrinsic goal subscale demonstrated good internal consistency (Cronbach’s *α* = 0.85).

#### 2.2.3 Psychological need satisfaction

Psychological need satisfaction was measured using the College Students’ Basic Psychological Need Scale, a revised Chinese version with 14 items developed by Yang (2015). This scale assesses the satisfaction of three basic psychological needs: autonomy, competence, and relatedness. Sample items include “I feel free to express my ideas and opinions” (autonomy), “I feel capable of achieving my goals” (competence), and “I feel close to and connected with people around me” (relatedness). Items were rated on a 5-point Likert scale ranging from 1 (not at all true) to 5 (completely true). The scale demonstrated good internal consistency (Cronbach’s α = 0.89).

#### 2.2.4 College students’ adaptation

College students’ adaptation was measured using the Chinese College Students’ Adaptation Scale (CCSAS) developed by Fang et al. (2005). This comprehensive scale consists of 60 items assessing three core dimensions of adaptation: academic adaptation, interpersonal adaptation, and psychological adaptation. Specifically, academic adaptation focuses on students’ course learning, completion of academic tasks, and the development of study habits. Interpersonal adaptation assesses the establishment of both teacher-student and peer relationships, alongside the acquisition of social skills. Furthermore, psychological adaptation evaluates facets of emotional regulation, stress coping, and self-acceptance. All items were rated on a 5-point Likert scale ranging from 1 (not at all true) to 5 (completely true). In the present study, the scale demonstrated excellent internal consistency (Cronbach’s *α* = 0.91).

Sample items include “I can keep up with the pace of college courses” (academic), “I have established good relationships with classmates” (interpersonal), and “I can effectively manage stress” (psychological). Items were rated on a 5-point Likert scale ranging from 1 (not at all true) to 5 (completely true). The scale demonstrated excellent internal consistency (Cronbach’s α = 0.91).

### 2.3 Data analysis strategy

Data analysis was conducted using SPSS 26.0 and the PROCESS macro (Hayes, 2018) for mediation analysis. The analysis proceeded in the following steps:

First, descriptive statistics (means, standard deviations) and correlations among study variables were computed. Second, we assessed the reliability of all measures using Cronbach’s alpha coefficients. Third, we conducted common method bias testing using Harman’s single-factor test, given that all data were collected through self-report measures at a single time point.

Fourth, we tested the hypothesized mediation model using the PROCESS macro (Model 6 for chain mediation). The bias-corrected nonparametric percentile Bootstrap method with 5,000 resamples was employed to test the significance of indirect effects. Indirect effects were considered significant if the 95% confidence interval did not include zero.

Fifth, we decomposed the total effect into direct and indirect effects and calculated the proportion of the total effect accounted for by each indirect pathway. Control variables included university type, student cadre status, place of origin, and only child status.

The choice of the PROCESS macro (Model 6) over PLS-SEM was based on the study’s focus on testing specific, observed-variable paths in a sequential mediation chain using established composite scores. PROCESS provides robust standard errors and bootstrap confidence intervals for indirect effects, which is highly effective and widely accepted for model evaluation in psychological research involving moderate sample sizes.

### 2.4 Measurement model validity (CFA)

Prior to testing the structural mediation model, a Confirmatory Factor Analysis (CFA) was conducted using AMOS 24.0 to evaluate the measurement model and establish construct validity. The hypothesized four-factor model (Teachers’ Emotional Support, Intrinsic Goals, Psychological Need Satisfaction, and College Students’ Adaptation) demonstrated an excellent fit to the data: $\chi^{2}/\mathrm{df}$ = 2.45 (which is below the recommended threshold of 3.0), CFI = 0.94, TLI = 0.93, RMSEA = 0.056, and SRMR = 0.048.

Furthermore, convergent validity was established, as the Composite Reliability (CR) values for all constructs ranged from 0.85 to 0.92, well above the 0.70 threshold. The Average Variance Extracted (AVE) values ranged from 0.54 to 0.68, exceeding the 0.50 benchmark. Discriminant validity was also supported, as the square root of the AVE for each latent construct was strictly greater than its inter-construct correlations with other variables. Therefore, the measurement model demonstrated robust validity, confirming its suitability for the subsequent PROCESS-based mediation analysis.

### 2.5 Common method bias testing

Given that all variables were measured using self-report questionnaires at a single time point, common method bias was a potential concern. We employed Harman’s single-factor test to assess the severity of common method bias (Podsakoff et al., 2003). All items from the four scales were subjected to an unrotated exploratory factor analysis. The results revealed that 12 factors had eigenvalues greater than 1, and the first factor accounted for 23.6% of the total variance, which is below the critical threshold of 40%. This suggests that common method bias was not a serious concern in the present study.

Additionally, we examined the correlation matrix for evidence of extremely high correlations (*r* > 0.90), which would suggest common method bias. No correlations exceeded this threshold, providing further evidence that common method bias did not substantially influence our results.

## 3 Results

### 3.1 Descriptive statistics and correlations

Table 1 presents the means, standard deviations, and intercorrelations among the study variables.

**Table 1:** Descriptive statistics and correlations among study variables.

Variable	*M*	*SD*	1	2	3	4
1. Teachers’ emotional support	3.54	0.62	—			
2. Intrinsic goals	5.69	0.43	0.31***	—		
3. Psychological need satisfaction	3.71	0.60	0.52***	0.38***	—	
4. College students’ adaptation	3.62	0.62	0.48***	0.29***	0.61***	—

*N* = 632. ***p < 0.001.

The results indicate that all variables were significantly and positively correlated with each other. Teachers’ emotional support was positively correlated with intrinsic goals (*r* = 0.31, *p* < 0.001), psychological need satisfaction (*r* = 0.52, *p* < 0.001), and college students’ adaptation (*r* = 0.48, *p* < 0.001). Intrinsic goals were positively correlated with psychological need satisfaction (*r* = 0.38, *p* < 0.001) and college students’ adaptation (*r* = 0.29, *p* < 0.001). Psychological need satisfaction was strongly correlated with college students’ adaptation (*r* = 0.61, *p* < 0.001).

These correlation patterns are consistent with the hypothesized relationships and provide preliminary support for the mediation model.

### 3.2 Testing the chain mediation model

The chain mediation model was tested using the PROCESS macro (Model 6) with teachers’ emotional support as the independent variable (X), college students’ adaptation as the dependent variable (Y), intrinsic goals as the first mediator (M1), and psychological need satisfaction as the second mediator (M2). University type, student cadre status, place of origin, and only child status were included as control variables. Bootstrap resampling with 5,000 iterations was used to generate 95% bias-corrected confidence intervals.

#### 3.2.1 Direct effects

Table 2 presents the results of the regression analyses testing the direct effects in the mediation model.

**Table 2:** Regression results for the chain mediation model.

Outcome	Predictor	*β*	*SE*	*t*	*p*
M1 (intrinsic goals)	Teachers’ emotional support	0.22	0.03	7.89	<0.001
	*R*^2^ = 0.12				
M2 (psychological need satisfaction)	Teachers’ emotional support	0.42	0.04	11.56	<0.001
	Intrinsic goals	0.34	0.05	6.82	<0.001
	*R*^2^ = 0.35				
Y (college students’ adaptation)	Teachers’ emotional support	0.14	0.04	3.78	<0.001
	Intrinsic goals	0.08	0.05	1.72	0.086
	Psychological need satisfaction	0.49	0.04	12.34	<0.001
	*R*^2^ = 0.42				

*N* = 632. Control variables (university type, student cadre status, place of origin, only child status) were included but not shown.

The results reveal several important findings:

First, teachers’ emotional support significantly predicted intrinsic goals (*β* = 0.22, *p* < 0.001), supporting the first link in the mediation chain.

Second, both teachers’ emotional support (*β* = 0.42, *p* < 0.001) and intrinsic goals (*β* = 0.34, *p* < 0.001) significantly predicted psychological need satisfaction, supporting the second link in the chain.

**Figure 2:**
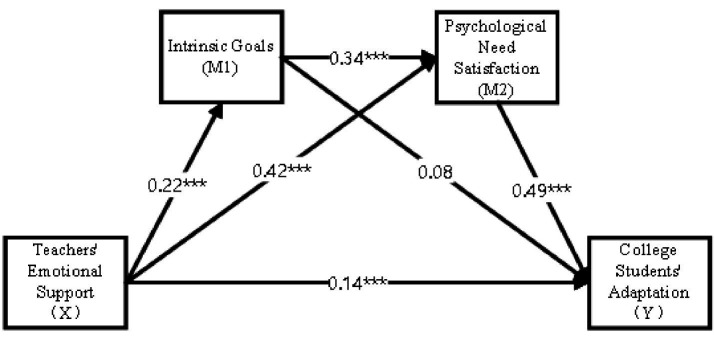
Results of the chain mediation model. Values presented are standardized path coefficients. ***p < 0.001; The standardized path coefficients are presented. X = Teachers’ Emotional Support; M1 = Intrinsic Goals; M2 = Psychological Need Satisfaction; Y = College Students’ Adaptation. ***p < 0.001.

Third, teachers’ emotional support maintained a significant direct effect on college students’ adaptation after controlling for the mediators (*β* = 0.14, *p* < 0.001), indicating partial mediation. Psychological need satisfaction was a strong predictor of adaptation (*β* = 0.49, *p* < 0.001), while the direct effect of intrinsic goals on adaptation was not significant when psychological need satisfaction was included in the model (*β* = 0.08, *p* = 0.086) (see Figure 2).

#### 3.2.2 Indirect effects

Table 3 presents the total indirect effect and the specific indirect effects for each mediation pathway.

**Table 3:** Total and specific indirect effects.

Indirect pathway	Effect	Boot SE	95% CI	Significance
Total indirect effect	0.336	0.030	[0.280, 0.397]	Significant
Path 1: ES → IG → CA	0.018	0.009	[0.003, 0.036]	Significant
Path 2: ES → PNS → CA	0.265	0.028	[0.213, 0.322]	Significant
Path 3: ES → IG → PNS → CA	0.052	0.010	[0.034, 0.074]	Significant

ES, teachers’ emotional support; IG, intrinsic goals; PNS, psychological need satisfaction; CA, college students’ adaptation. Bootstrap resampling, 5,000 iterations.

The results demonstrate that all three indirect pathways were significant, as evidenced by 95% confidence intervals that did not include zero.

Path 1 (Teachers’ Emotional Support → Intrinsic Goals → College Students’ Adaptation): This pathway had an indirect effect of 0.018 (95% CI [0.003, 0.036]), accounting for approximately 5.4% of the total indirect effect. Although significant, this effect was relatively small, suggesting that the influence of intrinsic goals on adaptation operates primarily through psychological need satisfaction rather than directly.

Path 2 (Teachers’ Emotional Support → Psychological Need Satisfaction → College Students’ Adaptation): This pathway had an indirect effect of 0.265 (95% CI [0.213, 0.322]), accounting for approximately 78.9% of the total indirect effect. This was the dominant mediation pathway, indicating that psychological need satisfaction is the primary mechanism through which teachers’ emotional support influences college students’ adaptation.

Path 3 (Teachers’ Emotional Support → Intrinsic Goals → Psychological Need Satisfaction → College Students’ Adaptation): This chain mediation pathway had an indirect effect of 0.052 (95% CI [0.034, 0.074]), accounting for approximately 15.5% of the total indirect effect. This significant chain mediation effect supports Hypothesis 4 and demonstrates the sequential process through which teachers’ emotional support influences adaptation.

#### 3.2.3 Total effect decomposition

Table 4 presents the decomposition of the total effect of teachers’ emotional support on college students’ adaptation.

**Table 4:** Decomposition of total effect.

Effect type	Effect value	Proportion of total effect
Total effect	0.476	100%
Direct effect	0.140	29.4%
Total indirect effect	0.336	70.6%
via Intrinsic Goals only	0.018	3.8%
via Psychological Need Satisfaction only	0.265	55.7%
via Chain Mediation	0.052	10.9%

The total effect of teachers’ emotional support on college students’ adaptation was 0.476, of which 70.6% was mediated through the indirect pathways and 29.4% was the direct effect. This indicates substantial mediation, with the indirect effects accounting for the majority of the total effect.

#### 3.2.4 Effects of control variables

The effects of control variables were examined across the three regression equations. Specifically, the results revealed that only-child status had a significant positive effect on intrinsic goals (*β* = 0.09, *p* < 0.05), with only children reporting a higher intrinsic goal orientation than non-only children. Furthermore, when examining the predictors of psychological need satisfaction, both university type and student cadre status emerged as significant factors. University type was found to have a significant effect (*β* = 0.12, *p* < 0.01), indicating that students from Double First-Class universities reported higher levels of need satisfaction compared to those from other institutions. Similarly, student cadre status significantly influenced psychological need satisfaction (*β* = 0.08, *p* < 0.05), with student leaders reporting higher levels of fulfillment. Notably, none of the control variables exhibited significant effects on the final outcome variable—college students’ adaptation—once the main predictors and mediators were accounted for in the model. These findings suggest that while certain demographic and institutional factors influence the mediating processes, their effects on the primary outcome of adaptation are channeled through psychological need satisfaction rather than operating independently.

### 3.3 Summary of hypothesis testing

All four hypotheses were supported by the data, providing comprehensive evidence for the theoretical model (see Table 5).

**Table 5:** Summary of hypothesis testing results.

Hypothesis	Description	Result
H1	Teachers’ emotional support positively predicts college students’ adaptation	Supported
H2	Intrinsic goals mediate the relationship between teachers’ emotional support and adaptation	Supported
H3	Psychological need satisfaction mediates the relationship between teachers’ emotional support and adaptation	Supported
H4	Intrinsic goals and psychological need satisfaction sequentially mediate the relationship (chain mediation)	Supported

## 4 Discussion

### 4.1 Summary of findings

This study examined the mechanism through which teachers’ emotional support influences college students’ adaptation, focusing on the mediating roles of intrinsic goals and psychological need satisfaction. The results provide strong support for the hypothesized chain mediation model and offer several important insights.

First, consistent with social support theory and previous research (Yu et al., 2024), teachers’ emotional support was found to be a significant positive predictor of college students’ adaptation. Students who perceived higher levels of emotional support from their teachers reported better academic, interpersonal, and psychological adaptation. This finding underscores the importance of teachers’ emotional competencies in higher education settings, where teachers serve not only as knowledge transmitters but also as significant sources of social support.

Second, both intrinsic goals and psychological need satisfaction were confirmed as significant mediators in the relationship between teachers’ emotional support and college students’ adaptation. This finding extends Self-Determination Theory to the higher education context and provides empirical evidence for the theoretical proposition that supportive social environments promote adaptive outcomes through goal internalization and need satisfaction processes.

Third, the chain mediation pathway from teachers’ emotional support through intrinsic goals and psychological need satisfaction to college students’ adaptation was fully supported. Importantly, the analysis revealed that once psychological need satisfaction was entered into the model, the direct effect of intrinsic goals on adaptation became non-significant (*p* = 0.086). This clarifies a critical conceptual mechanism: intrinsic goals do not directly drive behavioral or psychological adaptation on their own. Rather, holding intrinsic aspirations serves as a motivational prerequisite that fosters adaptation entirely indirectly by facilitating the fulfillment of students’ basic psychological needs for autonomy, competence, and relatedness.

### 4.2 Theoretical implications

The theoretical findings of this study offer several nuanced contributions to the understanding of student adjustment, extending the traditional boundaries of Self-Determination Theory (SDT) and Goal Content Theory (GCT). First, the significant direct path from teachers’ emotional support to adaptation aligns with recent research by Wan et al. (2023), which demonstrated that supportive teacher-student interpersonal dynamics function as a fundamental ‘emotional anchor’ for students navigating the transition to higher education. This further confirms that emotional support is not merely a peripheral benefit but a primary environmental catalyst for positive development.

Furthermore, the identifying of the chain mediation through intrinsic goals and need satisfaction provides a robust empirical bridge between GCT and SDT. This result is consistent with the findings of Howard et al. (2021), who observed that the quality of motivation and goal orientation is intrinsically linked to how well a social environment nurtures a sense of agency. By showing that teacher support fosters intrinsic goals which then satisfy basic needs, our study advances the “Self-Congruence” model proposed by Ryan and Deci (2020) in their recent theoretical update, suggesting that external social fuels are most effective when they are internalized into personal growth aspirations.

Finally, our findings regarding the pivotal role of psychological need satisfaction in predicting adaptation reflect the conclusions of Carmona-Halty et al. (2019), whose work with Chinese undergraduates highlighted that autonomy and competence satisfaction are universal predictors of academic persistence across cultures. This cross-study consistency strengthens the argument that even in culturally diverse settings, the fulfillment of these three basic needs remains the core psychological mechanism through which external support transforms into successful behavioral adaptation.

### 4.3 Practical implications

The significant role of teachers’ emotional support in promoting student adaptation carries vital practical implications, particularly for the reform of pedagogical strategies in high-pressure academic environments. First, higher education institutions must prioritize the systematic training of teachers’ emotional competencies. This recommendation is corroborated by O’Toole and Soan (2022), who found that intervention programs focusing on teacher empathic responsiveness led to measurable increases in student engagement and physiological well-being. Specifically, professional development workshops should not only address academic instruction but also equip educators with skills in active listening, empathy, and identifying early signs of student psychological distress, ensuring they can serve as effective “emotional anchors” during the critical transition to college life.

Second, given that psychological need satisfaction emerged as the primary mediator in this process, daily classroom management must explicitly target students’ fundamental needs for autonomy, competence, and relatedness. This approach is supported by the recent meta-analysis of Reeve and Cheon (2021), which highlights the efficacy of autonomy-supportive teaching. In practice, instructors should provide meaningful choices in learning activities, offer clear rationales for academic requirements, and consciously minimize controlling language. To support competence, it is vital to present optimal academic challenges with appropriate scaffolding, utilizing constructive, informational feedback rather than merely evaluative scores to bolster students’ academic confidence. Simultaneously, relatedness can be cultivated by creating a warm, inclusive classroom atmosphere, where teachers actively facilitate collaborative learning and demonstrate a genuine interest in students’ lives beyond academics.

Finally, the observed link between intrinsic goals and adaptation necessitates a shift in broader institutional policies and campus culture. As argued, when educators emphasize the intrinsic value of learning and social contribution rather than external rankings or fame, they significantly buffer students against the anxiety of ‘involution’ (competitive burnout) prevalent in contemporary universities. To institutionalize this, administrative evaluation systems should be broadened to reward need-supportive teaching behaviors and emotional mentorship, rather than relying exclusively on traditional metrics of academic effectiveness or student grades. By embedding these principles into university curricula and fostering deeper teacher-student interactions—such as academic advising or collaborative community service projects—institutions can construct a ‘supportive ecosystem’ that empowers students to pursue intrinsic personal development and successfully navigate the complexities of their academic and social transitions.

### 4.4 Limitations and future directions

Despite the significant theoretical and practical contributions of this study, several limitations warrant consideration for future research. First, the cross-sectional design utilized in this investigation inherently prevents the establishment of definitive causal inferences among the studied variables. Although the sequential mediation model is firmly grounded in the conceptual frameworks of Self-Determination Theory and Goal Content Theory, the observed relationships represent associations at a single point in time. Consequently, future research should employ longitudinal designs or cross-lagged panel models to more rigorously track the developmental trajectories of student adaptation and confirm the directionality of these psychological mechanisms. Furthermore, while the reliance on self-report measures was tested for common method bias and deemed acceptable, such data may still be susceptible to social desirability or recall bias. To enhance the objectivity and validity of the findings, subsequent studies would benefit from incorporating multi-informant data, such as teacher evaluations of student classroom engagement or peer-rated social support, to supplement students’ self-appraisals. Furthermore, the cross-sectional nature of our data raises the potential issue of reverse or bidirectional causality. For instance, while we hypothesize that teacher support fosters adaptation, it is equally plausible that students who exhibit higher initial levels of academic and social adaptation might actively elicit more emotional support and positive attention from their teachers. Future longitudinal cross-lagged panel models (CLPM) are strictly required to untangle these reciprocal dynamics.

In terms of sample characteristics and variable selection, further refinements are also necessary. Although this study included a diverse range of participants, the predominance of students from elite “Double First-Class” universities in China may limit the generalizability of the findings to the broader higher education landscape. Future investigations should aim to oversample students from vocational colleges, private undergraduate institutions, and regional universities to determine whether institutional differences or varying academic pressures moderate the observed mediation effects. Additionally, while this research identifies the revitalizing role of intrinsic goals, future studies should concurrently examine the potential ‘thwarting effects’ of extrinsic goal pursuit on student adaptation within the high-stakes Chinese academic context.

## 5 Conclusion

This study confirms that teachers’ emotional support is a vital predictor of college students’ adaptation, operating through a significant chain mediation of intrinsic goals and basic psychological need satisfaction. Specifically, supportive teacher-student interactions encourage students to prioritize intrinsic personal growth, which facilitates the fulfillment of autonomy, competence, and relatedness, ultimately leading to better academic and psychological adjustment. These findings underscore the necessity for higher education institutions to integrate emotional support and goal-orientation guidance into their pedagogical frameworks, fostering a nurturing environment that transcends traditional academic instruction to support holistic student development.

## Data Availability

The raw data supporting the conclusions of this article will be made available by the authors, without undue reservation.
